# Compare the clinical value of two minimally invasive approaches to locating radial nerve in the posterior humeral approach

**DOI:** 10.1186/s12891-023-06291-3

**Published:** 2023-03-13

**Authors:** Jin-Yi Feng, Wen-Bin Xu, Wu-Ji You, Gang Rui, Qing-Xiang Wang

**Affiliations:** 1 Department of Orthopedics, The First Affiliated Hospital of Xiamen University, School of Medicine, Xiamen University, Xiamen, China; 2 The Third Clinical Medical College, Fujian Medical University, Fuzhou, China; 3 Department of Anesthesiology, The First Affiliated Hospital of Xiamen University, School of Medicine, Xiamen University, Xiamen, China

**Keywords:** Radial nerve, Color Doppler ultrasonography, Posterior antebrachial cutaneous nerve, PACN, Humeral shaft fractures

## Abstract

**Purpose:**

To compare the clinical value between locating radial nerve (RN) guided by Color Doppler ultrasonography and posterior antebrachial cutaneous nerve (PACN) in the posterior humeral approach.

**Methods:**

The five fresh adult cadavers (ten upper arms) were selected to compare the two methods of locating the RN in the posterior humeral approach (guided by ultrasound and PACN) by measuring the operation time, the length of incision, and the area of subcutaneous free. And the comparison between the two groups was statistically analyzed by paired t-test.

**Results:**

The results of this study demonstrated that the length of incision and the area of subcutaneous free in the ultrasound group were smaller than that in the PACN group (P < 0.05), while the operation time was just the opposite (P < 0.05). However, after excluding the time of ultrasound location, the operation time in the ultrasound group was shorter than that in the PANC group, and the difference was statistically significant (P < 0.05).

**Conclusion:**

The RN can be quickly and safely exposed by both methods. The ultrasound approach requires a long learning curve, but is more minimally invasive and can help determine whether the intraoperative nerve is compressed by the plate. And the PACN method requires a longer incision and a wider area of subcutaneous free, while specialized equipment and professional training for surgeons are not required. In a word, these two methods have advantages and disadvantages, so they should be selected based on the exact situation.

## Introduction

Humeral fractures are common fractures in adults, accounting for about 7–8% of all fractures [1, 2], of which fractures of the shaft account for 20% of humeral fractures [3, 4]. With the development of the internal fixation technique, open reduction internal fixation (ORIF) has become the main treatment method. Although complications such as elbow stiffness are significantly decreased using ORIF, the consequent iatrogenic radial nerve (RN) injury is a topic that can never be avoided. It is reported that the incidence of RN injury caused by operative therapy is significantly higher than that caused by conservative therapy [5].

In the clinic, the damage caused by nerve injury to patients can easily lead to medical disputes. Therefore, how to quickly and accurately find the RN during the operation is particularly significant. Currently, some studies suggested that both approaches (guided by Color Doppler ultrasonography; guided by the posterior antebrachial cutaneous nerve, PACN) of locating the RN are considered to be quickly, safely and effectively in the clinical application [6, 7]. However, no studies have been reported to compare the clinical efficacy of these two approaches in locating the RN.

Hence, we performed this study to compare these two methods and to provide the latest evidence for clinical selection.

## Materials and methods

### Subjects

Ten upper extremities from five adult human cadavers (three male and two female) were acquired from the Department of Anatomy, Xiamen University. Prior to death, these cadaver specimens had no upper arm soft tissue damage, fractures, inflammation, tumors and other diseases, and the local skin and soft tissue anatomic structure were complete. In their lifetime, the donors had given their informed consent when they donated their bodies to the Department of Anatomy, Xiamen University.

### Exposure route and procedure

#### Guided by Color Doppler ultrasonography

**Fig. 1:**
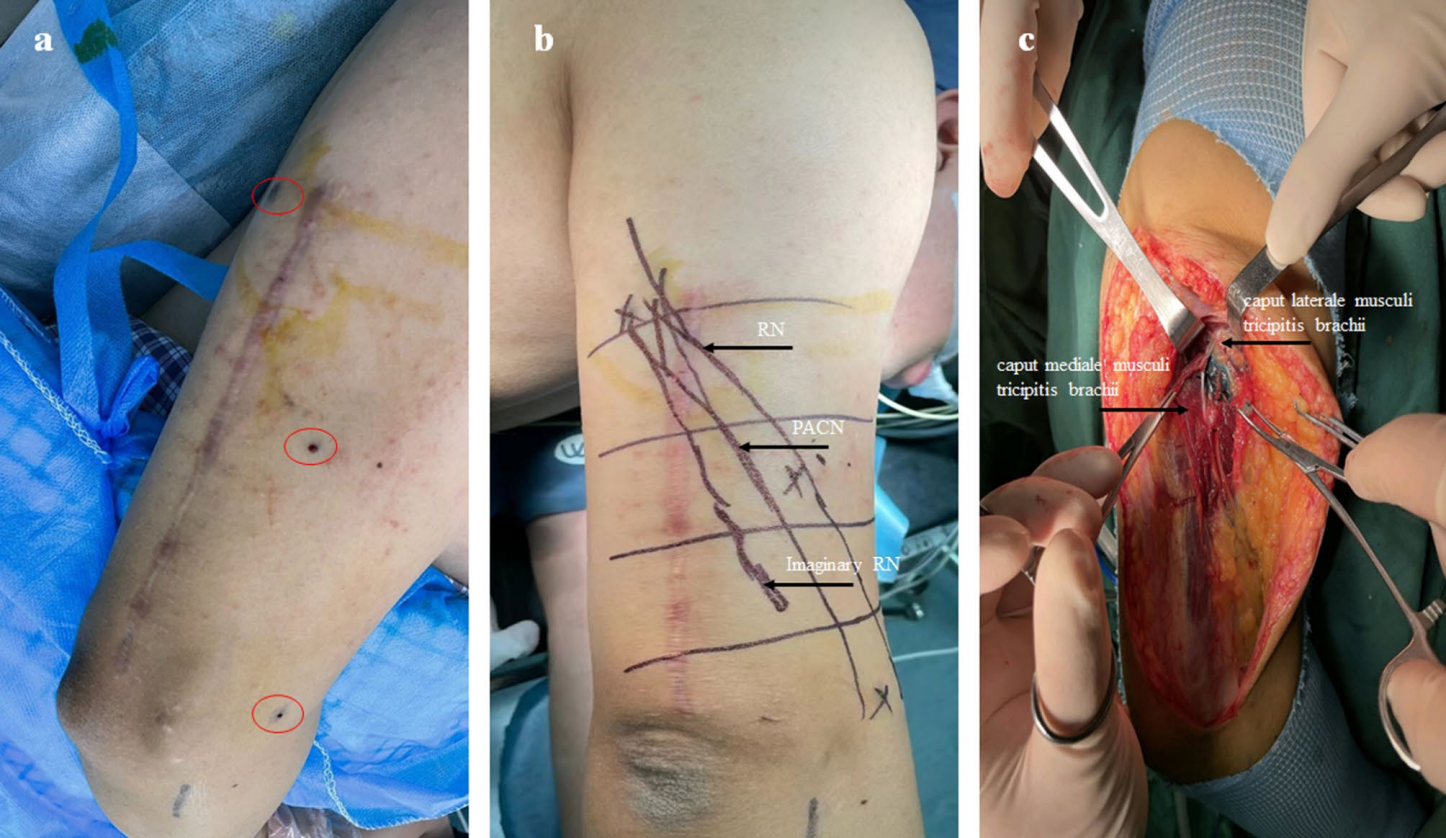
**a** Preoperative injection of methylthioninium chloride guided by ultrasound. **b** The body surface locations of the imaginary RN, PACN and RN were marked on the skin of the posterior upper arm; **c** Intraoperative image of the posterior upper arm

The linear array probe (6-12 MHz) was used to locate the RN in the lateral decubitus position. Then, methylthioninium chloride (0.5ml) was injected from three different ultrasound planes to mark the passage of RN (Fig. 1a), and corresponding marking lines were drawn on the body surface at the same time (Fig. 1b). Subsequently, the posterior median incision of the humerus was made, and the compartment between caput longum musculi tricipitis brachii and caput laterale musculi tricipitis brachii was separated from the proximal humerus, and the triceps tendon was cut longitudinally from the distal humerus. Finally, the caput laterale and caput mediale musculi tricipitis brachii were bluntly separated to find the RN according to the methylthioninium chloride (Fig. 1c).

#### Guided by the posterior antebrachial cutaneous nerve

**Fig. 2:**
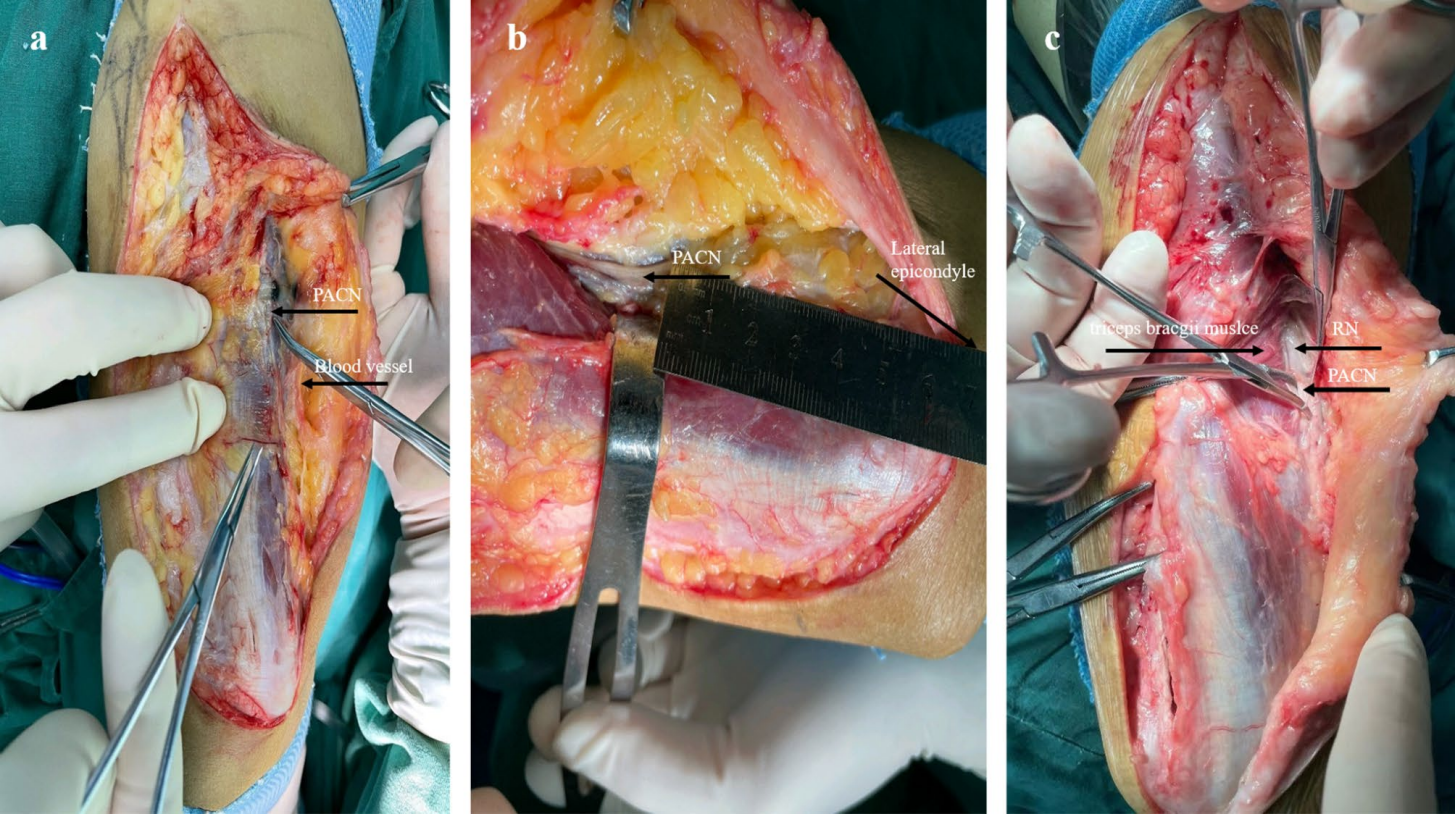
**a** Intraoperative image of the posterior upper arm shows the PACN and accompanying blood vessel. **b** Intraoperative image of the posterior upper arm demonstrates the PACN and lateral epicondyle. **c** Intraoperative image of the posterior upper arm shows the RN is identified by the PACN

The posterior median humeral incision was made to the level of the olecranon fossa in the lateral decubitus position (the distal incision is lateral to the outside). Then, the PACN accompanying the collapsed blood vessel can be seen by subepidermal anterolateral free (Fig. 2a). Subsequently, the subepidermal proximal free was performed (approximately 7–10 cm above the condylus lateralis humeri) (Fig. 2b) and then the deep fascia was cut. Finally, the RN could be found by careful free (Fig. 2c).

### Statistical analysis

Statistical analyses were performed using SPSS 23.00 software. Paired t-test was utilized to compare clinical outcomes between the two groups, such as operation time, length of the incision, and free subcutaneous area. Statistical significance was set at *p* < 0.05.

## Results

Five adult human cadavers were selected (three male and two female) and altogether ten arms were dissected and analyzed. The mean height of subjects was 172 ± 10.80 cm. The overall results were showed in Table 1.

**Table 1:** The overall results of two approaches of locating RN

	Operation time (min)	Operation time (min) (only RN exposure time)	Length of incision (cm)	Free subcutaneous area (cm^2^)
Ultrasound Group	28.600 ± 4.040	8.800 ± 1.790	16.020 ± 1.010	64.570 ± 4.090
PACN Group	11.800 ± 2.390	11.800 ± 2.390	19.680 ± 1.780	140.650 ± 17.230
t-value	21.000	9.487	4.893	12.403
p-value	0.000	0.001	0.008	0.000

PACN, posterior antebrachial cutaneous nerve; RN, radial nerve; min, minute(s); cm, centimeter

### Operation time

The mean operation time was 28.60 ± 4.04 min (including ultrasound location and RN exposure time) in the color Doppler ultrasound group, 8.80 ± 1.79 min (only including RN exposure time) in the Doppler ultrasound group, 11.80 ± 2.39 min in the PACN group. The mean operation time in the PACN group was remarkably lower than that in the color Doppler ultrasound group, and the difference was statistically significant (p < 0.05).

### Length of the incision

The mean length of the incision was 16.02 ± 1.01 cm in the color Doppler ultrasound group, and 19.68 ± 1.78 in the PACN group. The outcomes demonstrated that the mean length of the incision in the color Doppler ultrasound group was significantly better than that in the PACN group (p < 0.05).

### Free subcutaneous area

The mean free subcutaneous area in the color Doppler ultrasound group was 64.57 ± 4.09 cm^2^, while the area in the PACN group was 140.65 ± 17.23 cm^2^. The results indicated that the free subcutaneous area in the color Doppler ultrasound group was significantly smaller than that in the PACN group (p < 0.05).

## Discussion

The humeral shaft fractures are mostly spiral and comminuted fractures (unstable fractures), so conservative treatment is very likely to fail [8–10]. With the increasing sophistication of ORIF, more and more patients are opting for operative treatment to avoid the dysfunction of the shoulder and elbow. Currently, operative treatment has become one of the most common treatments for this type of fracture [11]. Despite innovations in techniques such as intramedullary nail and minimally invasive internal fixation (MIPO), RN injury is still inevitable [12–14].

At present, ultrasound-guided preoperative localization of RN and intraoperative exploration of nerve injury have gradually gained clinical recognition [15]. However, the learning curve of this method is relatively long [15]. Ultrasound-guided localization of RN often takes a lot of time if the operator lacks the relevant experience. According to our clinical work experience, it usually takes 15 to 20 min or even 30 min to locate the RN under ultrasound guidance. However, many senior surgeons believe that it is not difficult to quickly identify the RN by direct intraoperative anatomical localization, which is consistent with the literature report that the probability of the RN injury is negatively correlated with the seniority of the operator [16].

There is a certain difference between ultrasound-guided localization of the RN in cadavers and in patients, and usually localization in cadavers takes longer. Excluding the ultrasound localization time, it was found that the operation time in the color Doppler ultrasound group was significantly shorter than that in the PACN group, which reduced the probability of injury, improved safety, and enhanced the self-confidence of the operator during the operation. Furthermore, the results indicated that radial nerve localization by ultrasound can reduce the incision length and subcutaneous free area, and obtain better treatment with less trauma. With the widespread promotion of nerve block technology under the guidance of ultrasound, it will be widely used in clinical orthopedic surgery to help avoid nerve injury due to its advantages of simple equipment, bedside operation, and non-invasive [17–20].

Currently, there are no studies reported on the application of ultrasound-guided localization of the RN in secondary removal internal fixation, and the safety and effectiveness in the second operation need to be verified by further clinical studies. Meanwhile, one related clinical article excluded patients with old fractures and fracture nonunion, which indirectly indicates that the feasibility of ultrasound-guided localization of the RN in such patients has not been clinically validated [21].

**Fig. 3:**
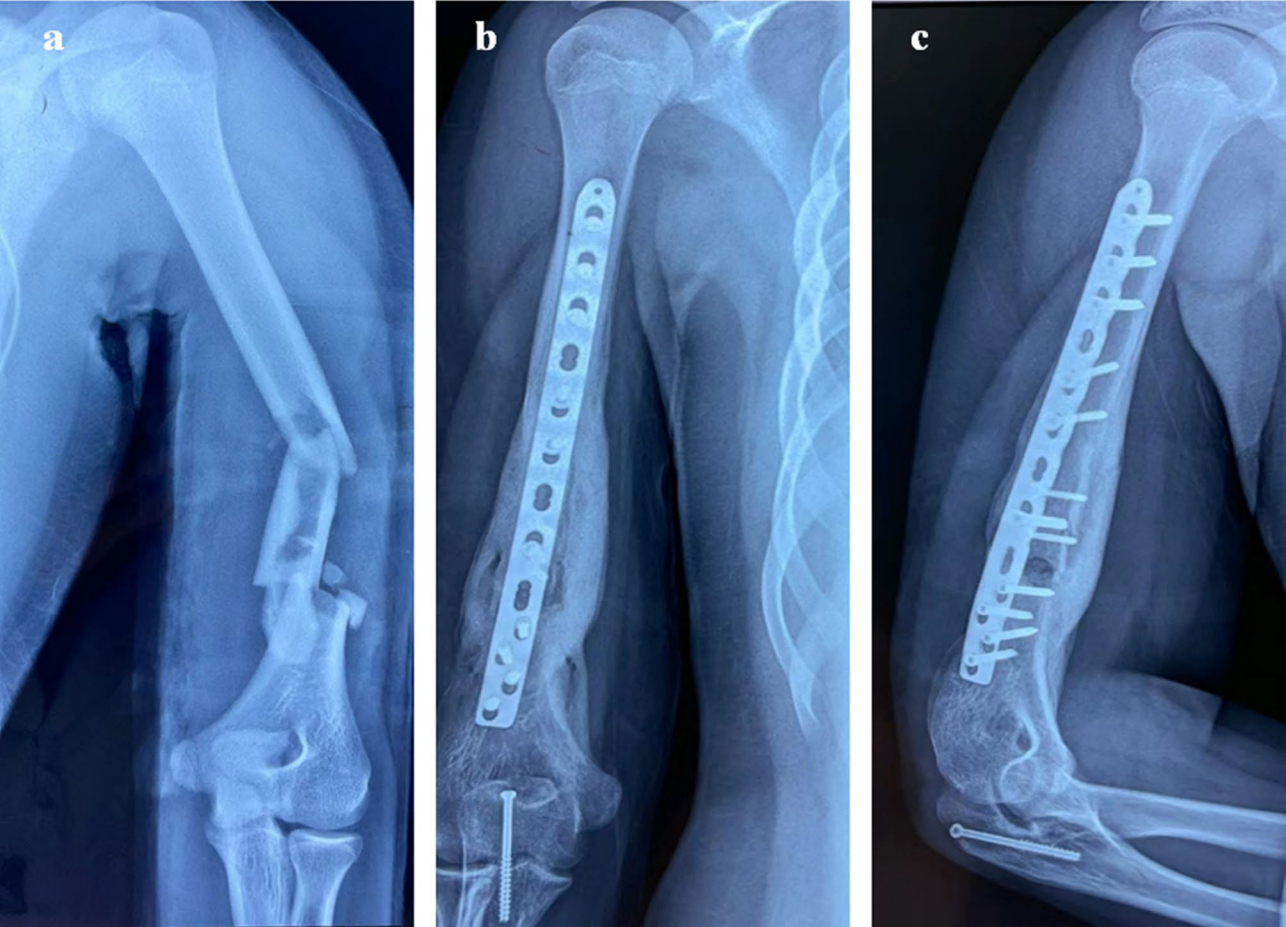
Radiographs of a patient with multi-segmental, comminuted humeral fracture. **a** Anteroposterior (preoperative). **b** Anteroposterior (postoperative infection). **c** Lateral (postoperative infection)

In our clinical work, a patient with a multi-segmental, comminuted humeral fracture (Fig. 3a) had to undergo secondary removal of internal fixation due to postoperative infection (Fig. 3b and c). Preoperative ultrasound showed that the RN could not be found due to hyperplastic scar and high-level echo of the plate. Therefore, the PACN approach was applied to localize the RN. This method can effectively avoid RN injury using only the surgeon’s hand but does not significantly increase the operation time. In addition, we routinely used the nonabsorbable suture as a marker in the muscle tissue on the surface of the RN during the primary internal fixation operation (humeral shaft fractures) using the plate (keeping the knot loose to avoid causing iatrogenic RN compression). When secondary operation was necessary for some reasons (infection, fracture nonunion, and failure of internal fixation), the RN could be quickly located by this special marker without any complications such as nerve injury or difficulty in finding it. However, further clinical studies are needed to verify this approach. The iatrogenic injury of the RN is highly correlated with the experience of surgeons. For experienced surgeons, it is not necessary to find the RN through ultrasound, PACN, and suture marking, but the prolongation of the operation time is inevitable and the risk of nerve injury remains high. However, if the RN is located by the above three approaches, even young surgeons also can find it safely and effectively.

As far as we all know, ultrasound-guided RN localization requires not only specialized ultrasound equipment, but also surgeons to participate in specialized ultrasound training, which has a certain learning curve. Moreover, in the process of ultrasound-guided localization, it is necessary to apply a special coupling agent on the skin surface of patients to facilitate observation. However, when methylthioninium chloride is injected by puncture, it cannot be strictly sterilized. Whether this will increase the risk of internal fixation infection, further studies need to be performed to validate. For the PACN localization method, advantages can be listed as follows: [1] the operation is simple, and the RN can be localized by simply expanding the free subcutaneous area; [2] the risk of infection is not increased; [3] it is convenient for primary hospitals to carry out; [4] no increase in healthcare costs.

## Conclusion

In conclusion, our findings indicate that despite differences in techniques, both PACN and ultrasound-guided localization can quickly and safely find the RN. The ultrasound method requires specialized equipment and professional training for surgeons (long learning curve), but the length of incision and the area of subcutaneous free are relatively small (minimally invasive) and can determine whether the nerve is compressed by the state during the operation. And the PACN approach requires a longer incision and a wider area of subcutaneous free, while specialized equipment and professional training for surgeons are not required. We believe that these two approaches have strengths and limitations, so they should be selected according to the exact situation.

## Data Availability

Please contact the corresponding author if data is required.

## Abbreviations

RN: Radial nerve
PACN: Posterior antebrachial cutaneous nerve
ORIF: Open reduction internal fixation
min: Minute
cm: Centimeter
MIPO: Minimally invasive internal fixation
